# In Vitro Evaluation of Accuracy and Reliability of Tooth Shade Selection Using Different Digital Instruments

**DOI:** 10.7759/cureus.52363

**Published:** 2024-01-16

**Authors:** Maaz Vohra, Amrutha Shenoy

**Affiliations:** 1 Department of Prosthodontics, Saveetha Dental College and Hospitals, Saveetha Institute of Medical and Technical Sciences, Saveetha University, Chennai, IND

**Keywords:** digital photography, dental photography, technology, scientific research, health

## Abstract

Introduction

Managing tooth shade is a significant challenge in aesthetic dentistry, especially for anterior restorations. Accurate shade assessment, combined with tailored treatment strategies and effective communication, is crucial. To improve the precision and reliability of aesthetic dental treatments, new shade-matching technologies have emerged. Current clinical methods for determining tooth shade utilize both visual assessments and instrumental techniques. The current study aimed to assess and compare the reliability and accuracy of four digital methods of tooth shade matching.

Materials and methods

This study utilized a 3D-printed resin upper arch model with tooth preparation done on tooth 11. An intraoral scanner was employed to scan and design the tooth, followed by the fabrication of 30 zirconia crowns using computer-aided design and computer-assisted manufacturing (CAD/CAM). The assessment of shade matching involved four digital photometric methodologies (group 1: twin flash + digital single-lens reflex (DSLR) camera (DT), group 2: ring flash + DSLR camera (DR), group 3: smartphone camera (SMART), group 4: intraoral scanner (IOS)) with Commission Internationale de l'Eclairage (CIEL*a*b*) values determined through Adobe Photoshop transformation. Accuracy (ΔE) was calculated and a specific shade using Vitablocs Mark II 3D-Master served as the standard. CIEL*a*b* data (where L = lightness, a and b = chromaticity coordinates) from four cohorts were analyzed in SPSS 26.0 for reliability, with intraclass correlation. The Kruskal-Wallis test and Spearman's correlation assessed reliability, while a one-sample t-test assessed accuracy, comparing values to clinical thresholds (p<0.05).

Results

The intraclass correlation revealed noteworthy variations in the L*, a*, and b* values, spanning from 0.730 to 0.994, 0.885 to 0.992, and 0.881 to 0.997, respectively. Intraoral scanners demonstrated high accuracy (ΔE = 5.8), while the SMART method showed the lowest precision (ΔE = 12.09). Twin flash with DSLR (TF+DSLR) and ring flash with DSLR (RF+DSLR) displayed comparable precision, with ΔE values of 10.90 and 10.97 respectively.

Conclusion

The smartphone exhibited the least precision, displaying notable discrepancies in all CIEL*a*b* metrics when compared to the manufacturer-specified shades. Conversely, the intraoral scanner demonstrated higher accuracy and reliability compared to the other groups, with no discernible variation in any of the CIEL*a*b* values from the manufacturer's standard.

## Introduction

One of the top aesthetic concerns and hardest things for dental professionals is managing shade [1]. An appropriate treatment strategy, an effective treatment method tailored to the patient's aesthetic apprehensions, and clear communication are pivotal. The assessment of tooth shade holds paramount significance in aesthetic dental restoration, especially in the anterior region [2]. In order to enhance the exactitude, replicability, and efficacy of aesthetic dental restorations, various novel technologies for shade matching have been developed. Presently employed clinical methods for ascertaining tooth shade in dental practice encompass both visual and instrumental approaches [3]. Owing to its simplicity and economical nature, visual identification of tooth shades remains the endorsed and extensively employed approach. Its principal advantage lies in its ability to corroborate consensus between the patient and clinician regarding the chosen shade. Nevertheless, the visual approach to shade assessment typically relies on diverse benchmarks, resulting in subjective outcomes, and primarily hinges on the efficacy of human visual perception [4]. To transcend the constraints inherent in the visual approach, which includes factors like environmental conditions and personal variations, instrumental shade determination was developed. Numerous academic references endorse the use of intraoral scanners (IOS), spectrophotometry, and cameras over-reliance on visual techniques [3,5]. Recently, the field of dentistry has embraced the integration of digital technologies aimed at enhancing clinical results, exemplified by the advent of IOS. Furthermore, in cosmetic dentistry, digital and smartphone cameras have seen extensive use. There is contention that a capture with a digital single-lens reflex (DSLR) camera could provide accurate shade measurements [6,7]. DSLR cameras, equipped with a variety of flash systems, enhance photography by offering customizable lighting. Through-the-lens (TTL) systems ensure correct exposure by measuring flash output based on camera settings. While built-in flashes are practical for occasional use, external flashes offer greater power and versatility. Twin flash (TF) and ring flash (RF) systems are commonly used in dental photography. The TF system, composed of two independent flash units on either side of the camera lens, improves depth and reduces harsh shadows in macro photography, providing even illumination and minimizing the need for additional modifiers. An RF, a circular-shaped flash unit around the camera lens, produces a distinct, even, and shadow-free light, often utilized in macro photography. With advancements in smartphone cameras and intricate technologies, many practitioners have adopted smartphones as their preferred tool for shade analysis. However, DSLRs provide better texture and image visualization, and recent intraoral scanners with built-in software use AI to evaluate the shade of scanned teeth. This necessitates a distinction between the aforementioned technologies. The color framework employed in dentistry, which categorizes a shade into hue, value, and chroma, is represented by the Munsell color space [8,9]. The L*, a*, b* color space, also known as CIEL*a*b*, is another commonly used system for shade matching. The delta E (ΔE) metric, used for assessing accuracy, serves as a numerical gauge to discern the color distinction between two objects, facilitating meaningful comparative analysis. As ΔE increases, so does the perceptible dissimilarity in color [10]. According to certain studies [11-13], the human eye cannot distinguish between different colors in a clinical setting until the ΔE value reaches 3.3. This underscores the necessity of using advanced technologies, especially in restoring anterior esthetic regions. With current evaluation technologies, shade matching has become a crucial part of dentistry. Many digital strategies are now being considered for this purpose. DSLRs with various flash systems have proven to be a handy method for matching tooth shades. With advances in intraoral scanning technology, many companies are now incorporating shade selection within the scanning protocol, thereby saving time and effort. The aim of the current study was to evaluate and compare four different digital tooth shade matching methods, and to assess the reliability and accuracy of these methods. The null hypothesis states that there would be no significant difference between the four methods in assessing the accuracy and reliability of shade selection.

## Materials and methods

The in vitro study conducted at the Department of Prosthodontics at Saveetha Dental College and Hospitals in Chennai, India, received institutional approval under the reference number SRB/SDC/FACULTY/22/PROSTHO/069. The sample size determination was based on statistical parameters derived from a previously published study [14], and the G*Power 3.1.9.3 software for Mac OS X® [15] was employed for this purpose. This enabled the utilization of 30 samples for the in vitro assessment of accuracy, as projected from the data using an independent samples t-test with a power threshold of 80%.

Sample preparation

**Figure 1:**
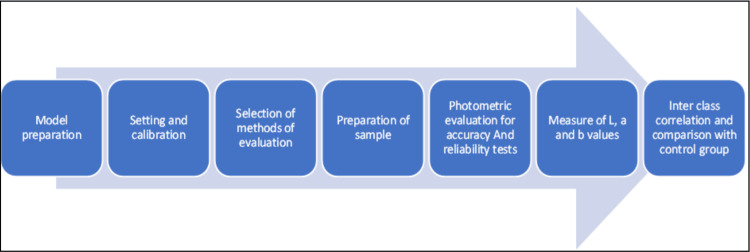
Flowchart depicting the workflow followed for shade matching.

The upper arch model, with tooth preparation done on tooth 11, was fabricated using a 3D-printed resin (Dionavi-P. Max UV resin, South Korea), spanning from the midpoint of the first molar to the midpoint of the first molar on the other side. An 18% gray hue resin was deliberately chosen to minimize light dispersion around the specimens. This 3D model was scanned using the 3Shape Trios scanner (3Shape, Niels Juels Gade 13, Copenhagen, Denmark) to design the central incisor (tooth 11). Subsequently, 30 milled zirconia crown prostheses were fabricated using computer-aided design and computer-assisted manufacturing (CAD/CAM) technology based on the obtained STL file. The samples were milled from Vitablocs (VITA Zahnfabrik, Bad Säckingen, Germany), covering nine distinct VITA 3D-Master hues. An overview of the entire study design is provided in Figure 1.

Assessment of shade matching

**Figure 2:**
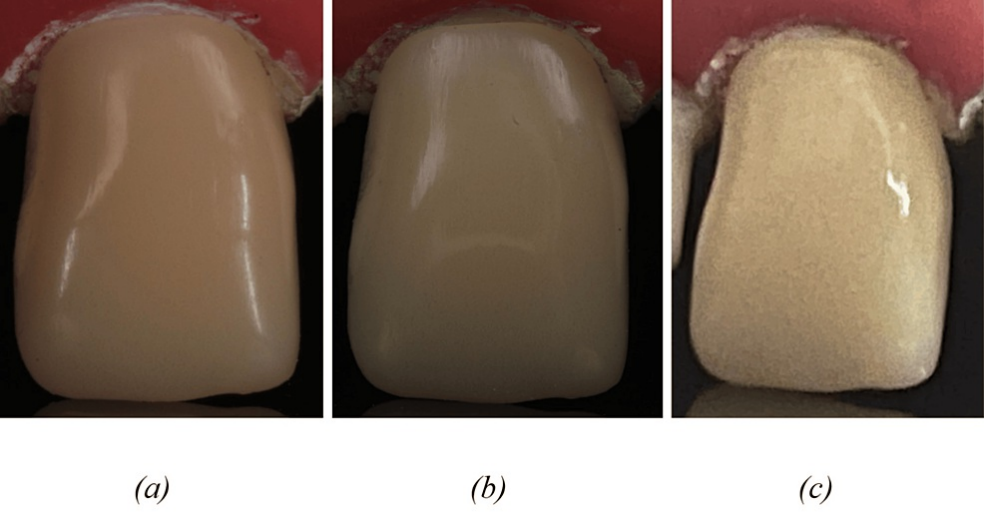
Images depicting shade using (a) TF+DSLR, (b) RF+DSLR, and (c) SMART. TF+DSLR: Twin flash + Digital single lens reflex camera; RF+DSLR: Ring flash + Digital single lens reflex camera; SMART: Smartphone camera.

Photographs were obtained in RAW format by employing four distinct digital photometric methodologies, which included a DSLR + TF, a DSLR + RF, a SMART, and an IOS (Table 1 and Figure 2).

**Table 1:** Characteristics of the interventional photometric equipments. TF+DSLR: Twin flash + Digital single lens reflex camera; RF+DSLR: Ring flash + Digital single lens reflex camera; SMART: Smartphone camera; IOS: Intraoral scanners.

Group	Equipment	Calibration
1	TF (Godox MF 12 k2) + DSLR (Canon 1500D with 100 mm macro lens)	Shutter speed-1/200, f29, ISO-100, magnification-1:3, white balance-Auto, distance-30 cm
2	RF (Godox ML-150)+ DSLR (Canon 1500D with 100 mm macro lens)	Shutter speed-1/200,f29, ISO-100, magnification ratio-1:3, white balance-Auto, distance-30 cm
3	SMART (Iphone 12)	f50, ISO-200, flash-Auto, magnification ratio-1:3, white balance-5000 K, distance-15 cm
4	IOS (3 shape Trios)	Manufacturer’s recommendations

After gathering the photographic data, the RAW files were sorted into four distinct folders, each labeled with a specific method name. The images were cropped and contrasted with the product's hue to determine the CIEL*a*b* values. Shade assessment was then conducted after the digital images were transformed into CIEL*a*b* values using Adobe Photoshop software 2020 (San Jose, California); these values were later tabulated and compared. The measurement point on each photo was identified, and the CIEL*a*b* system was used to produce and contrast values between each method. The central-most grid, which is the most focused and free of any reflections caused by the flash system, was utilized. Delta E was obtained using the following formula: ΔE*ab =[(ΔL)² + (Δa)² + (Δb)²]1/2 ΔE= Shade accuracy L* = lightness a*, b* = chromaticity coordinates According to the manufacturer's recommendations, the study established a specific shade using Vitablocs ® mark II 3D-master which was used as a standard for measurement. To convert shade measurements provided according to the VITA Zahnfabrik to the CIEL*a*b* system, a conversion table was employed [16].

Statistical analysis

CIEL*a*b* values data from the four study cohorts were gathered and organized in Google Sheets. Subsequently, this dataset was transferred into SPSS software version 26.0, developed by IBM Corp. (Armonk, NY, USA). Reliability was assessed based on two rounds of intraclass correlation. The evaluation of both the study and the established standard was carried out using the Kruskal-Wallis test and Spearman's correlation to ascertain the accuracy of the study variables. To compare the E values of the research cohorts with the clinical acceptability threshold (E = 6.8), a one-sample t-test was executed. Moreover, the assessment of CIEL*a*b* values between the study cohorts and the established benchmark was performed via the Kruskal-Wallis test, with a significance level set at p < 0.05.

## Results

Reliability and accuracy of measurements were obtained from four distinct test groups in comparison to a standard CIEL*a*b* value. During the assessment of measurement consistency across the groups, intraclass correlation revealed statistically significant variations between the groups (p<0.05), ranging from 0.730 to 0.994, 0.885 to 0.992, and 0.881 to 0.997 (Table 2).

**Table 2:** Interclass correlation of CIEL*a*b* measurements within the study cohorts. TF+DSLR: Twin flash + Digital single lens reflex camera; RF+DSLR: Ring flash + Digital single lens reflex camera; SMART: Smartphone camera; IOS: Intraoral scanners.

	Group	Mean	Lower limit	Upper limit	P-value
L* value	TF+DSLR	0.915	0.478	0.961	0.002*
	SMART	0.936	0.348	0.974	0.002*
	RF+DSLR	0.848	0.228	0.936	0.005*
	IOS	0.994	0.984	0.996	0.765
a* value	TF+DSLR	0.963	0.674	0.981	0.323
	SMART	0.942	0.571	0.972	0.345
	RF+DSLR	0.968	0.768	0.984	0.546
	IOS	0.994	0.974	0.996	0.786
b* value	TF+DSLR	0.986	0.892	0.994	0.865
	SMART	0.992	0.935	0.996	0.876
	RF+DSLR	0.999	0.991	1.000	0.578
	IOS	0.939	0.994	1.001	0.977

The accuracy of each test group concerning the standard CIEL*, a*, and b* values was assessed using the Kruskal-Wallis test and Spearman correlation at a 95% confidence interval. The results revealed significant differences in L values across all groups, except for IOS, when compared to the set standard. These findings demonstrate varying degrees of accuracy across the test groups, except for IOS. When comparing the E values across all groups with the clinically acceptable threshold (E = 6.8), notable statistically significant discrepancies were observed. In particular, the E value for the IOS was recorded at 5.8, and none of the L, a, or b metrics displayed any statistically significant deviation from the shade specified by the manufacturer (p < 0.01). Conversely, the DSLR + TF, DSLR + RF, and SMART methods exhibited E values exceeding the clinical acceptance threshold (E > 6.8). Notably, SMART exhibited the lowest level of accuracy (E = 12.09), while TF+DSLR and RF+DSLR demonstrated comparably high levels of precision (E = 10.90 and 10.97) (Table 3).

**Table 3:** Comparison of mean ∆E values for each group. TF+DSLR: Twin flash + Digital single lens reflex camera; RF+DSLR: Ring flash + Digital single lens reflex camera; SMART: Smartphone camera; IOS: Intraoral scanners.

Mean ∆E	Comparison groups	Mean difference between the standard and test groups	SD
E =10.90	TF+ DSLR	9.815	2.30
E=12.09	SMART	27.106	2.74
E=10.97	RF+DSLR	8.755	2.38
E=5.8	IOS	2.496	1.9

The SMART yielded the least accurate outcomes, contrasting with the IOS, which emerged as the most precise technology.

## Discussion

The present study delves into tooth shade matching in aesthetic dentistry, a subject of paramount importance for dental professionals. It is an in-depth investigation that compares the efficacy and reliability of four digital shade-matching methods. The study's findings have direct implications for clinical practice, guiding dental professionals in selecting the most accurate and reliable shade-matching methods for aesthetic dental restorations. The current investigation employed four distinct study groups: (1) TF + DSLR (Canon 1500D with 100 mm macro), (2) RF + DSLR (Canon 1500D with 100 mm macro), (3) SMART, and (4) IOS, compared with Vitablocs ® mark II 3D-master, which was used as a standard for measurement. These study groups were selected for the investigation as they are the most commonly used instruments for documentation in dentistry and are readily available to students and practitioners. In all of these cohorts, the L, a, and b values obtained through the CIEL*a*b* model displayed impressive intraclass correlation coefficients (with r > 0.7, r > 0.8, and r > 0.8, respectively). Notably, despite the inclusion of two repetitions, the reliability assessment yielded highly favorable outcomes. In essence, the process of tooth shade selection proved to be exceedingly dependable with the utilization of all the equipment employed in this investigation. SMART exhibited the lowest level of accuracy when compared to the manufacturer's designated shade, as all CIEL*a*b* parameters (comprising L, a, and b values) resulted in the null hypothesis being rejected [17]. Furthermore, this specific group demonstrated the most pronounced ΔE (E = 19.98) when juxtaposed with all other methodologies. ΔE, often denoted as color differential, quantifies the discernible difference in color between two entities. A higher ΔE value suggests a more noticeable discrepancy in perceived hue quality [18]. Unlike a DSLR camera equipped with various TF setups, Sampaio CS et al. identified that the iPhone 12, as well as the DSLR camera paired with a ring flash, demonstrated the highest E values. This study provides significant insights into the precision of tooth shade selection using digital cameras along with diverse techniques and equipment. Notably, the research did not include a comparative evaluation between utilizing a DSLR camera with a ring flash and polarizing filter or using an adaptable smartphone camera with the same filter [17,19].

According to the findings of this investigation, the application of an IOS showcased the utmost accuracy. Importantly, none of the CIEL*a*b* metrics (L*, a*, and b* values) displayed any notable disparity from the shade specified by the manufacturer, as indicated by p-values of 1, 0.5, and 1, respectively. Additionally, this specific group recorded the most minimal E value among all cohorts (E = 5.96), comfortably falling within the expected clinical acceptance range (E < 6.8). In sharp contrast, the RF+DSLR and TF+DSLR methods exhibited elevated E values (10.97 and 10.90, respectively), both surpassing the anticipated clinical acceptance range. This highlights that only the IOS group achieved a visually pleasing result in shade determination throughout this study [20].

Acknowledging the constraints within the present in-vitro study, it is crucial to consider various factors like the presence of saliva, lighting conditions, and the polychromatic nature of natural teeth when applying these techniques intraorally. One of the limitations is the lack of diversity in camera gear; the DSLR camera used in this study was a crop sensor camera with entry-level features, making it accessible to beginners. In the case of smartphone photography, the images obtained are usually warped, which could have significantly altered the results. These limitations may impact the generalizability of the findings to a broader context, as the data generated from a specific cohort might be constrained by these factors. Given these limitations, further research endeavors are essential to refine data interpretation when employing diverse instrumental methodologies. Furthermore, the field of dental photography should consider incorporating a gray card to harmonize image tones using more refined software, consequently augmenting shade congruence. The fusion of various methodologies and a learning curve for dental professionals is imperative for achieving the highest level of precision in outcomes.

## Conclusions

Based on the above findings, the smartphone camera demonstrated the least precision, whereas the IOS exhibited the highest level of accuracy. In situations where precise color matching is crucial, especially in cosmetic dentistry procedures, the use of IOS can be employed. This necessitates training and education among dental professionals to enhance their skills in digital dentistry.
